# Sequential assembly *in vivo* of the bacteriophage P22 genome translocation channel

**DOI:** 10.64898/2026.09.03.749273

**Published:** 2026-09-09

**Authors:** Huaxin Yu, Jian Yue, Taehyun Park, Chunyan Wang, Muyuan Chen, Jun Liu, Ian J. Molineux

## Abstract

Bacteriophage P22 ejects four internal proteins into infected cells to assemble a channel for genome translocation across the cell envelope. However, how these proteins cooperate to build the trans-envelope channel remains enigmatic. Using *in situ* single-particle cryogenic electron microscopy, we determined near-atomic *in vivo* structures of P22 infection intermediates, revealing how the tail hub protein gp10 templates the sequential assembly of three internal proteins gp7*, gp20 and gp16 into a ∼50-nm trans-envelope channel that extends from the virion into the host cytoplasm. Functional analyses explain why phage DNA can be detected in supernatant fluids during infection by channel-defective mutants. Our structures also provide a mechanistic basis for the long-standing observation that a P22 virion lacking gp16 can be complemented, extra-cytoplasmically, by a co-infecting virion lacking gp20, thereby allowing the genome in the gp16-deficient particle to enter the host cytoplasm.

## Full Text Availability

The license terms selected by the author(s) for this preprint version do not permit archiving in PMC. The full text is available from the preprint server.

